# Impact of Visual Impairment and Dual Processing on Gait in Older People

**DOI:** 10.1093/geroni/igaf122.2563

**Published:** 2025-12-31

**Authors:** Maura Mlecko, Desirae Sandridge, Evie Burnet

**Affiliations:** College of William and Mary, Williamsburg, Virginia, United States; College of William and Mary, Williamsburg, Virginia, United States; College of William and Mary, Williamsburg, Virginia, United States

## Abstract

Research linked visual impairment to cognitive decline in older people; it has not assessed how visual impairment affects cognitive measures, specifically dual processing tasks involving cognition and gait. To investigate whether visual impairment impacts dual processing performance, shown by velocity, stride width, and double support percent. 70 independent living residents aged 71 to 98 were assessed for visual acuity using the Snellen Test. Participants were split into normal vision (48) and people with visual impairment (22). People with visual impairment were defined as presenting or best-corrected visual acuity less than 20/40 in the better eye. They walked at their typical pace on a sensor-embedded mat and repeated this while naming animals beginning with a specific letter for dual processing. A t-test was run with SPSS with significance p < 0.05. Differences between normal vision and individuals with visual impairment were insignificant in gait velocity (p = 0.083) and stride width (p = 0.266) with normal gait, but significant in these measures during a dual processing task. Velocity was significantly faster (p = 0.016) in normal vision compared to people with visual impairment. Stride width was significantly greater (p = 0.022) in people with visual impairment compared to normal vision. There was no significant difference in double support percent in either condition. Visual impairment impacted dual processing performance shown by a slower velocity and greater stride width while performing a dual processing task. Further research is needed to determine if corrective lenses mitigate the effects of visual impairment on dual processing tasks.

